# Rapid Induction and Maintenance of Remission in Refractory Ulcerative Colitis with Ustekinumab

**DOI:** 10.3390/diseases7040055

**Published:** 2019-09-28

**Authors:** An-Yu Chen, Helieh S. Oz

**Affiliations:** 1 Gastroenterology, Lexington Clinic, Lexington, KY 40504, USA, achen@lexclin.com; 2 Department of Medicine, UK Medical Center, Lexington, KY 40536, USA

**Keywords:** inflammatory bowel disease, ulcerative colitis, biologics, ustekinumab, therapeutics

## Abstract

Ulcerative colitis is a chronic debilitating disease characterized by relapsing in intestinal inflammation and ulcers with no available cure. This is a clinical case report of a 52-year-old female patient with 30 years history of left-sided chronic ulcerative colitis controlled with standard of care (mesalamine and azathioprine) which subsequently relapsed and developed into active refractory ulcerative colitis. The patient became unresponsive to her medications including different forms of mesalamines and did not respond favorably to any of the other current therapies. Numerous attempts to stabilize her condition with immunosuppressants, steroids, probiotics, antibiotics, mesalamines, and various biologic agents failed to improve her clinical symptoms, and the patient was being considered for colectomy. As the last resort, modified therapy was prescribed with ustekinumab, a non-selective, anti-IL12/23 p40 monoclonal antibody. This medication has not been yet approved for use in ulcerative colitis patients. In this clinical case we report the efficacy of ustekinumab to rapidly induce and maintain remission of the severe chronic ulcerative colitis in the patient. To the best of our knowledge, this is the first report of utilizing ustakinamub therapy for rapid induction in an active refractory ulcerative colitis patient resulting in complete remission for over one year.

## 1. Introduction

### Inflammatory Bowel Disease

Ulcerative colitis and Crohn’s disease are chronic debilitating diseases, characterized by recurrentintestinal inflammation. Despite the advent of biologics and other advancements in treating and stabilizing complications, there is still no cure for chronic inflammatory bowel diseases (IBD). Over 50-60% of these patients become refractory to standard of care available treatments with severe side effects including hepatotoxicity, ulcerogenic potential (form wounds), and severe blood disorders, as explained previously [1,2,3]. Also, about 20–30% of patients withdraw fromanti-tumor necrosis factor (TNF) therapy due to primary non-response to the drug, and a further 30–40% of patients become non-responsive within the first year [4]. Ulcerative colitis is a complex immune disease, with altered normal gut microbiota (dysbiosis) and increased gut permeability to gram (−) microbial toxins. Genetic variability in subjects is also considered as a possible factor. Other factors involved are increased reactive oxygen species released mainly from inflammatory aggregates [5,6]. More than half of these patients may not reach remission with currently available conventional or biologic treatments. Ulcerative colitis mostly affects the distal colon and rectum, with ulceration restricted to the mucosal layer, and rarely the pathology can advance into the terminal ileum. While the use of biologic therapies has provided a big step in improving the condition of IBD patients, a significant proportion of these patients remain refractory, with an urgent need for new and effective therapeutic modalities.

## 2. Materials and Methods

### 2.1. Bioethics

Ethical approval and consent for the case report are not applicable. All personal identifiers and information were blocked from this paper including patient’s name, address, phone and ID numbers before data were provided for preparation of this paper. No identifier is used in the report.

### 2.2. Informed Consent

Informed consent was obtained from the patient after providing opportunity for questions.

### 2.3. Colonoscopy Procedure

Instrument: Video Olympus CF Q180AL Colonoscope, ASA class: Level-II. Images were produced using Infinite Software Solutions Inc, www.infinitesoftsol.com & www.md-reports.com.

Indication (2010): Blood in stool, active ulcerative colitis, universal chronic ulcerative colitis. Indication (2018): Active ulcerative colitis with diarrhea.

Patchy active ulcerative colitis persisted while the patient was on a maximal infliximab therapy (10mg/kg q 4wk).Anser™ IFX (Infliximab) was an assay to measure both drug level and antibody levels to Infliximab in serum during therapy of IBD patients.Anser™ IFX measures serum infliximab levels of <1.0 μg/mLand antidrug antibodies of <3.1U/mL. Anser™ is indicated to have high sensitivity, specificity, and accuracy [7].

## 3. Background: Case Presentation

**Figure 1:**
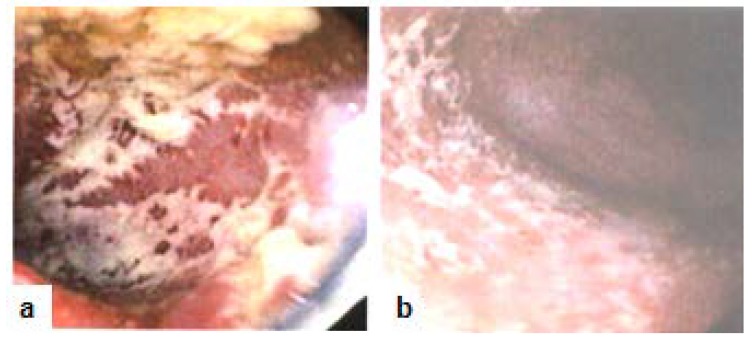
Colonoscopy (2010) with image representatives. (**a**) Rectum: Ulcerative colitis; (**b**) Sigmoid colon: Ulcerative colitis.

The patient is a 52 year old female, non-smoker, social alcohol user with a 30 years history of primarily left-sided colitis (Figure 1a,b, 2010). The patient was seen by her community primary care physician and was treated at various times with prednisone, several brands of mesalamine (e.g., Asacol HD^®^ or Lialda^®^ as dictated by insurance formularies), azathioprine (Imuran^®^), metronidazole (Flagyl^®^), and loperamide hydrochloride (Imodium^®^). Following multiple flare-ups with worsening symptoms which required a series of hospitalizations (2015), she was referred to the gastroenterologist (AC) for additional care management. The patient was being seen by primary care prior to this referral. The patient was started on golimumab (Simponi^®^), but after the fourth injection she was determined to be a primary non-responder, since her symptoms had deteriorated to pan-colitis. However, the levels of golimumab were not checked before.The patient experienced serial hospitalizations (2016) during which time she was treated with intravenous steroids, budesonide (Entocort^®^), rifaximin (Xifaxan^®^), metronidazole, probiotics VSL#3^®^, and mesalamine. On a follow-up visit after a hospitalization, (Remicade^®^, 5mg/kg/8wks) was initiated along with azathioprine(about 6 months later, in 2017). Her symptoms then improved temporarily, but soon relapsed with pan-colitis flares requiring repeated hospitalizations.Anser IFx^®^ test revealed no antibody titers (0) with low infliximab level (6.1 μg/mL). Infliximab dosage was then increased (to 10mg/kg/4wks) in conjunction with a short course of budesonide (Uceris^®^) during 2017. This led to about six months of symptomatic improvement during which she was once treated for *Clostridium difficile* (*C diff*) infection.

**Figure 2:**
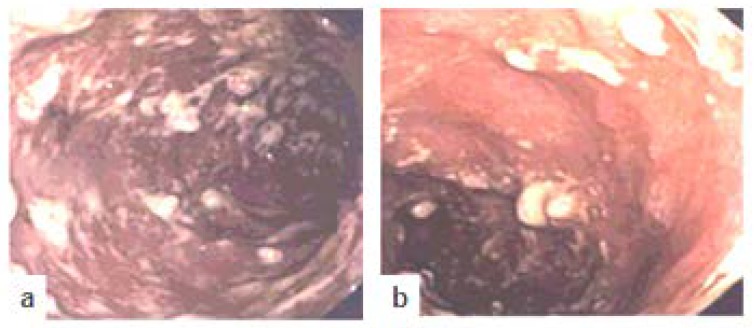
Colonoscopy (2018) with image representatives. (**a**) Transverse colon: Ulcerative colitis; (**b**) Descending colon: Inflammation, pseudopolyps. Inflammatory response with pseudopolyps and ulceration has been extended to the upper portion of the colonic tissues.

Unfortunately, her symptoms again worsened (2018), and a colonoscopy along with multiple biopsies revealed active refractory ulcerative colitis (Figure 2a,b, 2018).Therefore, patient was started on vedolizumab (Entyvio^®^) as primary agent for therapy. However, the patient became intolerant to vedolizumab and developed severe reactions to infusion (nausea, vomiting, diarrhea, and fever), possibly as a result of an allergic or hypersensitivity reaction. Thus, infusion therapy had to be discontinued after five sessions. At this stage, colectomy was considered and discussed with the patient.

While the patient was scheduled to visit the colorectal surgeon, a trial therapy with ustekinumab (Stelara^®^) was utilized as the last resort. One week after initial ustekinumab IV infusion, the patient reported a significant improvement of symptoms.Remission was then maintained with modified ustekinumab subcutaneous self-injection every sixweeks combined with azathioprineand the combination therapy acted effective with complete resolution of previous symptoms. It is anticipated that combination therapy to address multiple pathways of the inflammatory cascade prior monotherapy trials were ineffective.

Steroids were subsequently tapered and discontinued.Succeeding physical and laboratory markers (2019) have revealed complete symptomatic response to the treatment with ustekinumab. Currently, over one year after initiation of ustekinumab treatment, the patient is symptom-free with normalized laboratory markers including CBC, CMP, CRP, and has resumed her normal activities including full-time factory work.It is of interest that so far, the patient has been feeling well and unwilling to undergo repeat colonoscopy examinations to confirm mucosal healing.

## 4. Discussion

Ustekinumab (Stelara^®^), a non-selective anti-IL12/23 p40 monoclonal antibody, which has not yet been approved for use in ulcerative colitis patients by the FDA in the U.S.A. For this patient, a modified version of therapy was then designed to improve efficacy of regiments for the ulcerative colitis. The patient was prescribed ustekinumab subcutaneous self-injections every six weeks instead of a routine eight week regimen, as is utilized for Crohn’s disease. Physical and laboratory markers have revealed complete symptomatic response to ustekinumab treatment.Ustekinumab is an immune-modulator and a non-selective human anti-interleukin 12 and IL 23 p40 monoclonal antibodywhichis FDA-approved only for the use in Crohn’s disease, psoriasis, and psoriatic arthritis.Tofacitinib, another biologic, is an FDA-approved treatment for ulcerative colitis in patients. However, tofacitinib was not prescribed in this patient due to her insurance policy’s limitation.Ustekinumab is indicated for the treatment of adult patients (>18 years old), and those who have failed or become intolerant to immunomodulators (steroids, or anti-tumor necrosis factor (TNFα)). Ustekinumab appears to have a swift and lasting effect with potential for mucosal healing and systemic anti-inflammatory response with no known immunogenicity [8,9,10,11], in contrast to TNFα blockers. Ustekinumab may be preferred over anti-TNF therapy in older patients with increased risk for infections and malignancy, as concurrent immunosuppression may not be required due to its low immunogenicity [12]. In addition, after the firstintravenous injection, it is followed by subcutaneous self-administration. Ustekinumab has been used since November 2016 to treat moderately to severely active Crohn’s disease in adults with partial response or tolerance to the standard therapy with TNFα blockers [9,10]. Ustekinumab is used in combination with methotrexate, 6-mercaptopurine, azathioprine, or steroids, which may not affect the safety or efficacy of ustekinumab. Ustekinumab is not approved for use in pregnancy or breastfeeding; it may pass through the placenta or milk. Possible side effects are including, an increased risk of infections from a reduced ability to mount an immune response, as well as reactivation of latent infections which involves an increased risk of infections. Serious microbial infections include TB, viral, and fungal diseases. Crohn’s disease patients can develop anal abscesses, gastroenteritis, ophthalmic herpes, pneumonia, and meningitis caused by *Listeria monocytogenes*. Symptoms are chills, cough, fever, shortness of breath, weight loss, and pain.Patients are required to be tested for possible infectious agents before starting the regimen.Those patients who are predisposed to genetic variation in the proteins IL12 and IL-23 are at a higher risk for disseminated (systemic) infectious diseases such as *Salmonella, Mycobacteria*, and *Bacillus Calmette-Guerin* (BCG). Other possible infectious and inflammatory diseases are appendicitis, cholecystitis, cellulitis, diverticulitis, pneumonia, sepsis, osteomyelitis, and viral complications such as gastroenteritis and infectious complications such as gastroenteritis and urinary tract infections. Hypersensitivity: In case of serious allergic response, it is required to stop the medicine as follows; angioedema and anaphylaxis symptoms, episodes of fainting sensation, weakness, swelling of face, eyelids, tongue, throat, chest tightness, or skin rash.

Malignancies: Ustekinumab may increase the risk for neoplasia by decreasing immune response activity. Patients >60 years of age, or with prolonged immunosuppressant therapies, and on ustekinumab, should be monitored carefully for the symptoms of non-melanoma skin cancer. Reversible posterior leukoencephalopathy syndrome, a neurological disorder with an unknown origin, may affect the brain and cause fatality. Symptoms are headache, seizures, confusion, and vision problems.

Vaccination: Live vaccines are not recommended in patients using ustekinumab. Prior to initiating therapy, patients should receive all required immunizations, but not after being treated. In addition, non-live vaccines received during treatment may not cause appropriate immune response [13].

On October 9, 2018, a limited study (eight weeks) on induction data from the phase III UNIFI trial was presented at the American College of Gastroenterology (ACG) Annual Scientific Meeting 2018 [11]. The phase III trial was a double-blind, randomized placebo-controlled, multi-center, with a single intravenous (IV) infusion dose of ustekinumab. Ustekinumab caused clinical remission at eight weeks in 154 (16%) patients with moderate to severe ulcerative colitis (*n* = 961). These adult patients had refractory response to biologic (50%) or standard of therapies. Chief secondary outcomes included ratio of those in clinical response, endoscopic and historical mucosal healing, and health-related quality of life IBD questionnaire scores, which were all significantly improved at week eight fromtherapies with application of ustekinumab, compared with those in the placebo arm, indicating ustekinumab as a potential effective treatment for ulcerative colitis [11]. Twenty Crohn’s and three ulcerative colitis patients, of whichfourteen patients had psoriasic form lesions and nine had eczematic form lesions due to TNF blocker, were treated with ustekinumab in an IBD trial. Ustekinumab was reported to completely resolve psoriasic form lesions in 12 (85.7%) patients, two (14.3%) demonstrated partial response, 14 (61%) had complete digestive response, 10 (44%) had partial digestive response, and two (9%) failed to positively respond. Therefore, ustekinumab is recommend as a drug of choice in these patients [14]. While this paper was under review (September 4, 2019), the European Commission has recently approved the expanded use of ustekinumab (Stelara^®^) by Janssen’s, for the treatment of moderately to severely active ulcerative colitis in the European Union [15].

## 5. Conclusions

In conclusion, over one-year remission has been obtained in this patient with non-responsive active persistent chronic ulcerative colitis after treatment with modified ustekinumab therapy. This, along with the safety profile as documented in Crohn’s patients and in other autoimmune diseases such as arthritis and psoriasis, proves a potential for ustekinumab as an effective therapy for patients with non-responsive persistent chronic ulcerative colitis. Additional clinical investigations are required to determine the long-term beneficial effects of ustekinumab in ulcerative colitis as well as Crohn’s patients.
